# Parents and clinicians: partners in perinatal bereavement research –experiences from the International Stillbirth Alliance Conference 2017

**DOI:** 10.1186/s40900-018-0137-8

**Published:** 2019-02-01

**Authors:** Rachel Rice, Daniel Nuzum, Orla O’Connell, Keelin O’Donoghue

**Affiliations:** 1 Pregnancy Loss Research Group, Irish Centre for Fetal and Neonatal Translational Research (INFANT), University College Cork, Cork, Ireland; 2 School of Applied Social Studies, University College Cork, Cork, Ireland; 3 Department of Obstetrics and Gynaecology, University College Cork, Cork University Maternity Hospital, Wilton, Cork, Ireland; 4 Cork University Maternity Hospital, Wilton, Cork, Ireland

**Keywords:** Stillbirth, Patient engagement, Bereavement care, Pregnancy loss, Conference, Patient partnership, Training and education

## Abstract

In recent years, there has been a global call to reduce the numbers of preventable stillbirths and increase public awareness about the incidence and impact of pregnancy loss. The lived experiences of bereaved parents have much to contribute to developing the research agenda and clinical care in pregnancy loss. The multidisciplinary Pregnancy Loss Research Group (PLRG) based at the INFANT Centre at University College Cork and Cork University Maternity Hospital, has an established practice of active engagement and participation of patient members. This partnership provided the catalyst to model a similar collaborative approach between clinicians, researchers and bereaved parents when the PLRG was successful in their bid to host the International Stillbirth Alliance (ISA) annual conference in 2017. Over 400 hundred delegates from around the globe attended the conference, of which one quarter were bereaved parents.

Establishing a culture of collaboration, support and mutual respect in the field of pregnancy loss, requires scientists, clinicians and parents to be brought together so each can be informed by the other in the efforts to prevent stillbirth and improve bereavement care. As part of ISA 2017 conference, a sub-committee of staff and parents was established to ensure that the voice of parents could contribute to the research agenda and developments in clinical and bereavement care. A creative workshop specifically for parents, followed by a parent assembly were organised to facilitate this. Remembrance activities, organised by the parent committee, were central to the conference and actively engaged in by parents, clinicians and researchers.

This commentary, written collaboratively by a parent, a chaplain, a bereavement and loss specialist midwife and a consultant obstetrician, gives voice to this experience, identifying four key messages that arose from our reflection on the conference. These include; the value of active partnership between clinicians and patients, the use of creativity as a unifying expression of grief and as a means to facilitate learning, the value of collaboration with global stakeholders in raising awareness about stillbirth, and the importance of facilitating meaningful patient/public engagement in scientific research. The potential for education and learning opportunities are also explored, highlighting the connection between parents, researchers and clinicians as central stakeholders in the prevention of stillbirth and in improving bereavement care.

## Plain English summary

The practice of patients as partners in clinical research has been a commitment of the Pregnancy Loss Research Group (PLRG) based at the INFANT Centre at University College Cork and Cork University Maternity Hospital (CUMH). In September 2017, the PLRG hosted the International Stillbirth Alliance Conference in Ireland. Over 400 delegates attended the conference, of which one quarter were bereaved parents. From the outset, parents who had experienced stillbirth and pregnancy loss were involved at every level of the organisation and delivery of this international scientific conference. A sub-committee of parents and staff was established to focus specifically on the participation of parents at the conference. The scientific programme was organised to ensure equal and meaningful participation of parents, clinicians and researchers. Particular attention was given to the voice of parents. Recognising that research, practice and policy needs to be informed by the experience of parents, a workshop and assembly specifically for parents attending the conference were organised to ensure that their wisdom and insights were valued and would contribute to future research. Remembrance activities drew on the skills and creativity of parents and healthcare professionals together. Hundreds of heart shaped mementoes were handmade by clinicians and parents for the conference as a powerful symbol of remembrance for stillborn babies around the world. This commentary written collaboratively by a parent, a chaplain, a bereavement and loss specialist midwife and a consultant obstetrician, highlights the importance of patients as active participants in scientific research and the key role they play in responding to the global efforts to prevent stillbirth and improve bereavement care. It suggests four key messages that arose from reflection on the conference. These include; the value of active partnership between patients and clinicians, the use of creativity as a unifying expression of grief and as a means to facilitate learning,, the value of collaboration with global stakeholders in raising awareness about stillbirth, and the importance of facilitating meaningful patient/public engagement in scientific research.

## Introduction

The role of patients as partners in clinical research has grown from one of paternalistic recipients of medical care, to one of engaged partners and invested stakeholders [1]. In the field of obstetrics, it is a sad reality that 20 % of pregnancies end in miscarriage and one in two hundred births end in stillbirth. As a result, many clinician and patient interactions revolve around the challenge of communicating very difficult and sad news [2]. Naturally, parents are left with questions concerning the circumstances of their loss and many become motivated to support efforts to improve care and investigations into pregnancy loss and perinatal death [2, 3]. Building on the growing move to develop an increasingly patient led approach to research studies, the lived experiences of bereaved parents have much to contribute to developing the research agenda and clinical care in pregnancy loss [4]. Bringing this experience to the research table is now a recognised ethical standard in patient and public research involvement [1, 5].

In recent years, there has been a renewed global call to re-double the efforts to reduce the numbers of preventable stillbirth and increase public awareness about the incidence and impact of perinatal death [6, 7]. The International Stillbirth Alliance (ISA) is a global collective of healthcare professionals, researchers and bereaved parents “working towards stillbirth prevention and improving bereavement care” [8]. The annual conferences of the ISA are scientific in nature, with global experts engaged in disseminating the most up to date research. With a considerable scientific focus, the capacity of bereaved parents to engage meaningfully has been limited.

The Pregnancy Loss Research Group (PLRG) based at the INFANT Centre at University College Cork and Cork University Maternity Hospital (CUMH), has an established practice of active engagement and participation of parent/patient members alongside the multidisciplinary clinical and research team [9]. This partnership provided the catalyst to model a similar collaborative approach between clinicians, researchers and bereaved parents when the PLRG was successful in their bid to host the ISA annual conference in 2017. Over 400 delegates (academics, clinicians, allied health professionals and bereaved parents), attended the conference, with one quarter of this number being bereaved parents.

This commentary, written collaboratively by a parent, a chaplain, a specialist bereavement midwife and a consultant obstetrician, gives voice to the experience of collaborative engagement between bereaved parents, clinicians, scientists and researchers and offers a critical reflection on the lived experiences of bereaved parents and clinicians working together in the sensitive and challenging area of stillbirth.

## Main text

### What we set out to do

The organising committee for ISA 2017 was made up of members of the pregnancy loss research group, the National Perinatal Epidemiology Centre and representatives from Féileacáin (the Stillbirth and Neonatal Death Association of Ireland). Although ISA is a scientific conference, there was unilateral support and commitment from the committee that parents would be involved in the organisation of the conference. A parent sub-committee was established - comprising of six parents and three full time staff members with the brief to focus particularly on the parental aspects of the conference.

The first meeting of the parent sub-committee was a significant one where new boundaries and a working relationship was developed between staff members and bereaved parents. Although the staff members were known to each of the bereaved parents, the parent members of the group had not met each other previously. While the common experience of pregnancy loss had brought each of the parents to the table, it was important that the group formed an equal partnership with staff in order to become a working group for the conference. It took a while to navigate this new relationship. The nature of contact between staff and parents at the time of pregnancy loss is uniquely intimate, and often what is most remembered by parents. As the group evolved we discussed the shifting boundary from care-giver and care-receiver, to a relationship based on partnership and equality and the change in dynamic of this for everyone. However, as planning proceeded, the benefits of this collaboration became very clear as the challenge of what to do and how to do it, required expertise from both parents and staff.

The local organising committee was committed to ensuring that conference registration for parents was as affordable as possible. From the outset, it was important to ensure that parents could participate in the same way as any other delegate attending the conference.

One of the parents remarked that they were “*struck by the inter-connectedness of everyone who would be attending*” and later reflected that “*research in this area cannot happen without parents who are willing to share their experiences of stillbirth and pregnancy loss. Parents cannot hope for better outcomes in future pregnancies without research in this area. Doctors, midwives and multi-disciplinary teams cannot continue to provide the best care without parents to learn from and evidence based research to inform their practice. Each and every delegate was as important as and to each other, and somehow we needed to create spaces and events that would reflect that*.”

### What we did

As we began to brainstorm ideas, having ways to remember and honour the lives of stillborn babies felt very important. Being conscious of the many people around the world who might not be able to attend the conference, it was decided to open an on-line register where families could register the name of their stillborn baby. Health professionals could also register the name of a stillborn baby they had cared for and each of these baby’s names would be included as part of the remembrance activities during the conference. The on-line register also facilitated the opportunity to represent the global reality of stillbirth which enabled a strong sense of inclusion at the conference, a point which many delegates later remarked upon. A dedicated social media twitter account for the conference was also utilised by a global audience before and during the conference, all of which contributed to raising international awareness.

**Fig. 1:**
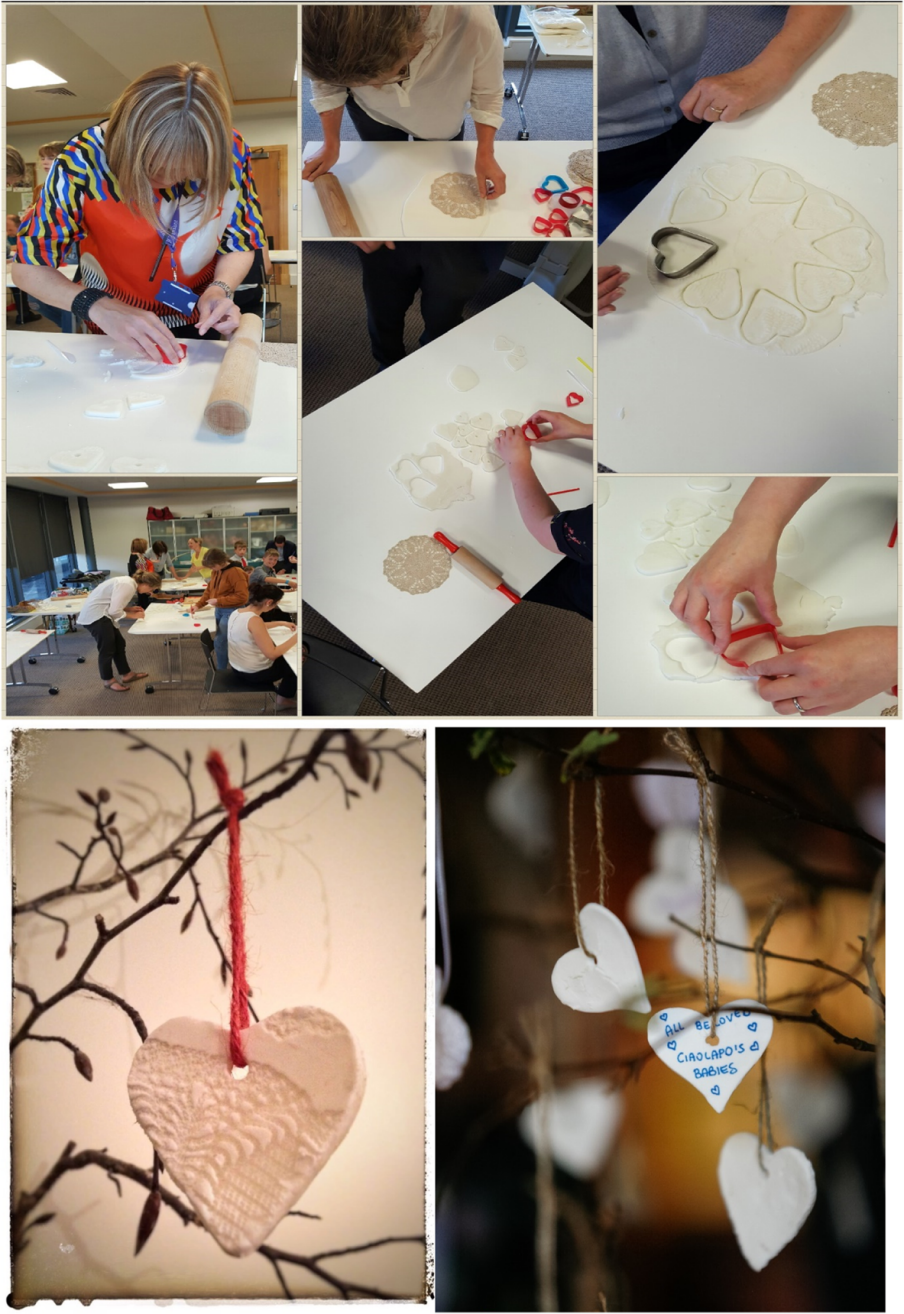
Making heart shaped mementoes – parents and clinicians

Having some way of appropriately representing these names at the conference became a much debated issue. Funding was a challenge and despite efforts to try and secure finances, we realised we needed to draw on some of the artistic talent and creativity among our group. The somewhat ambitious idea of making a heart shaped memento upon which a baby’s name could then be written, was decided. Little did we realise that in a very short space of time, nearly 400 baby names would be submitted online in advance and almost 150 more registered during the conference itself. With volunteers from the local organising committee and staff members we gathered as an eclectic group to make 600 clay hearts for the conference (Fig. 1).

This collaborative effort was significant for a number of reasons. The ‘creating’ of mementos somehow seemed to be the most natural fit with the creating of memories so often associated with pregnancy loss. Somehow the very embodied experience of losing a baby seemed best represented by using our own hands in this creative way. From a staff perspective, providing care to parents at the time of a stillbirth is also very ‘hands on’ and the engagement from staff who helped in making the hearts was representative of this.

For some parents in our group, it was the first time they had been back to the maternity hospital since their pregnancy loss. It was during the making of the hearts, that conversations began to naturally emerge about stories of stillbirths and loss, and memories were shared. It was no surprise that it was often the little things people remembered that were really significant at the time of their own baby’s stillbirth. The simplicity of a midwife clearing the lift so that one parent didn’t have to face anyone as she went from scan room to the ward, was a reminder that the small things really do matter at a time of real devastation. This was a theme that arose again and again at the conference itself. As the evening progressed and staff began to join ‘the production line’, one could not but be struck by the hearts somehow beginning to represent the very essence of what the conference was about. This enterprising and shared hands-on creative activity brought parents, pathologists, midwives, obstetricians and chaplains together in a very natural way and in so doing, symbolised the reason we were all ultimately involved.

Once dry, writing the names of babies on each heart required further evenings together in CUMH. As staff recalled the names of babies they had cared for and inscribed them on the hearts – it was a reminder that the impact of stillbirth isn’t just confined to parents. The poignancy of seeing so many names on the hearts from around the world was certainly not lost on any of us, but somehow kept us energised and motivated for the task ahead (Fig. 1).

**Fig. 2:**
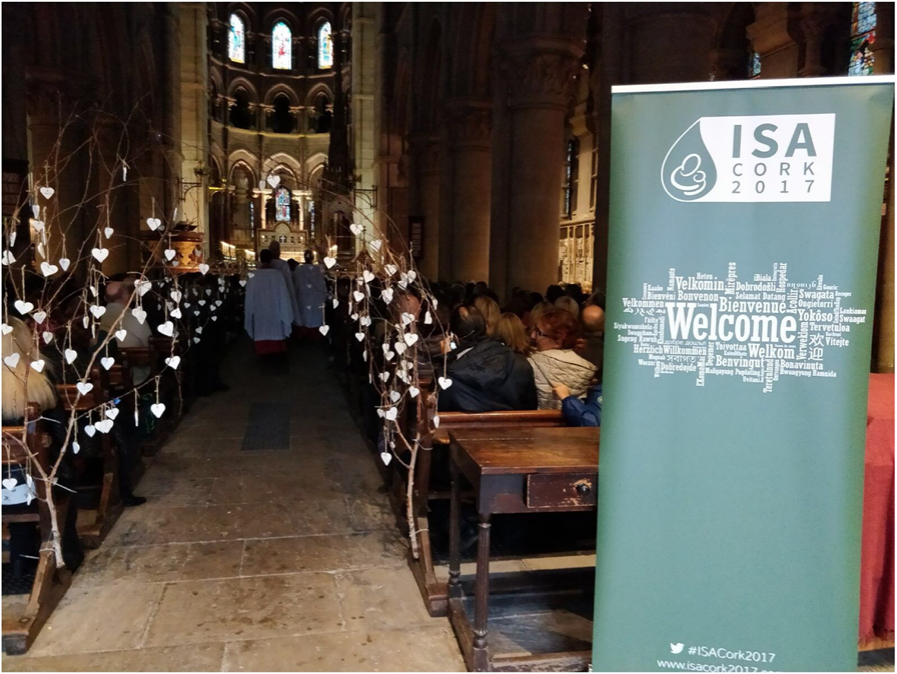
Remembrance service

**Fig. 3:**
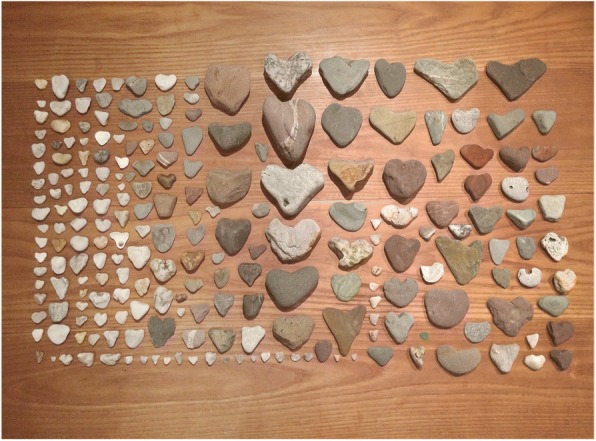
Heart shaped stones

A Service of Remembrance specific to the conference took place on the second evening, and as we began to plan for this service, it was decided that the hearts would be hung around the Cathedral, visible to all who would attend. With some novel engineering, (a 6 am handover of tree branches from chaplain to a parent with a large van among our group), each heart was strung late one evening in the Cathedral ready to be hung on the braches before the service began (Fig. 2). The parent group also chose to use heart-shaped stones as mementos for delegates to take away from the conference; symbolising the uniqueness and permanence of loss. As photographs of heart shaped stones that were collected on beaches around Ireland were posted on social media and twitter, packages began to arrive with heart shaped stones from around the world, offering support and encouragement for the conference (Fig. 3).

We were also conscious that the experience of being at the conference was likely to be quite intense and sad for bereaved parents, but also perhaps for clinicians and researchers. Providing an accessible “Quiet Room” for people to take some time away from the conference programme and for their own personal reflections, felt important. This space was decorated by the parent group and kindly hosted by two volunteers, who for many years have been providing a knitting service to the hospital for the babies of the pregnancy loss service. Parents and delegates were invited to spend time reading carefully chosen reflections or to inscribe a baby’s name on a heart shaped memento and hang it on a tree, or to simply sit in silence in the company of others. The committee also ensured that all delegates were aware that the bereavement team were available to give support should this be required at any stage during the conference.

Funding was secured to bring the nationally acclaimed “Amulet” exhibition by Marie Brett to the conference [10]. A previous collaborative project between bereaved parents and three Irish Maternity hospitals, this sound and visual interactive artwork aims to shed light on the often hidden world of infant loss. It felt appropriate to host this exhibition at the conference so that the voices and experiences of Irish women who had experienced stillbirth were represented in the powerfully unique and interactive way that this artwork allows for.

The importance of research, practice and policy in relation to stillbirth needing to be informed by the voice and experience of parents, was acknowledged by all on the organising committee. The idea of an Assembly where parents attending the conference could develop a collective statement on their thoughts for the future in this regard felt important.. Much discussion took place as to how best facilitate this. In preparation for the assembly, a creative workshop, facilitated by an Art Therapist and professional facilitator among our parent group, was devised. The purpose of the workshop was to provide parents with a facilitated space where art was used to help represent experience and to articulate learning and questions arising from the conference. It was decided that the workshop and assembly would run sequentially.

**Fig. 4:**
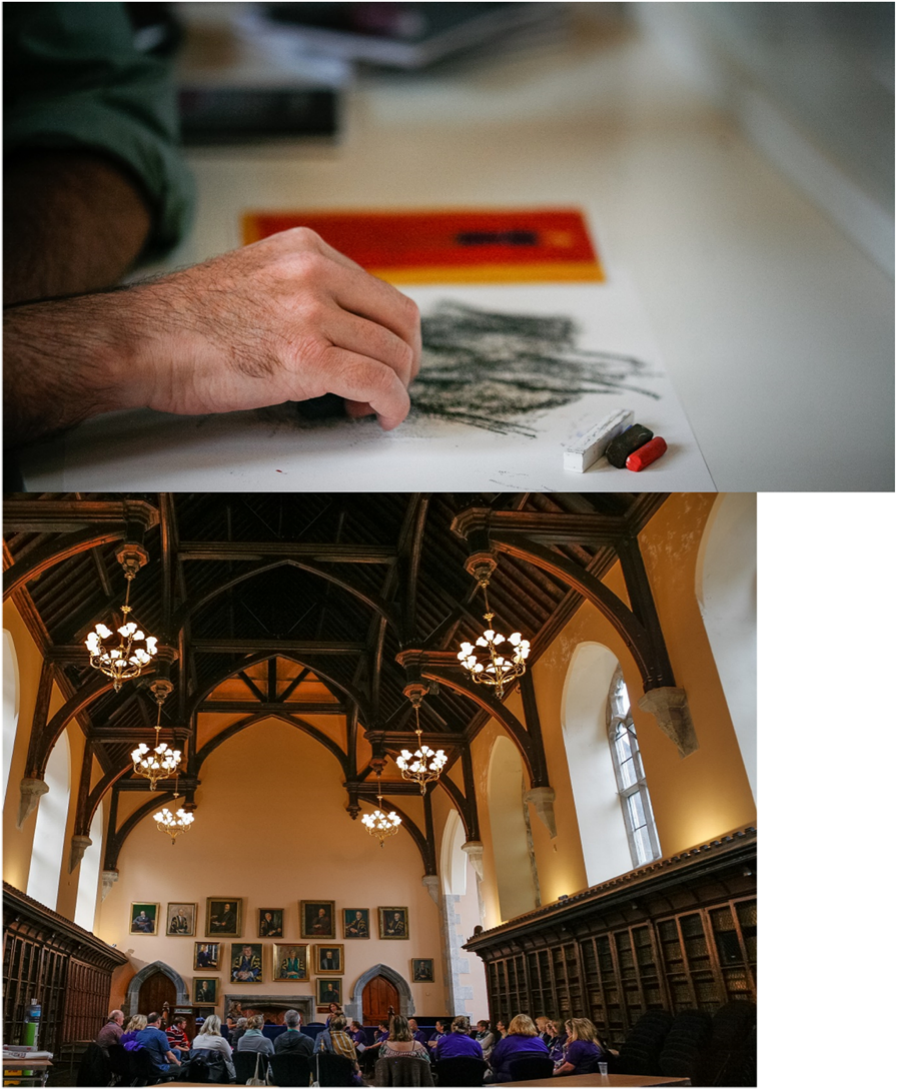
Parent workshop; “exploring the grief experience” and parent assembly

Having sufficient professional expertise to hold such a workshop was really important to ensure it would be a safe space for participants and we were fortunate that this expertise existed among our parent and staff committee. The use of art and image making are recognised powerful tools to help process people’s experience, and in the words of one of our parents, sometimes “when there are no words”, creative processes such as image making provide an alternative means to help people represent and articulate their experience [11] (Fig. 4). However, there was learning in this workshop for all of us: particularly how quickly people can be brought back to their own experience and stories of loss when given the space. It was important that we worked in small groups and there were sufficient facilitators in each group so parents were supported in what they made and then shared.

Moving from this workshop into the Assembly, parents were invited to think about what they would like to see happen in future research and practice in relation to stillbirth. Parents were asked to work in small groups and develop a collective statement on their thoughts in this regard. Parents were clearly energised about this and the suggestions and recommendations made were both considered and informed having listened to the research presented over the 2 day conference. Bagley et al., suggest that one of the challenges of patient and public involvement is that public contributors might be perceived to have an overly narrow or self-serving agenda [1]. This was not the experience of this conference. The interest and commitment of the parents bereaved through stillbirth was not self-serving, rather, they were there, offering their views and experience as advocates for a greater public good. Our hope as a committee was that being given a voice to influence policy, practice and a future research agenda would be a meaningful and empowering experience for parents. A submission has since been made on behalf of the parents who participated in the assembly to the implementation group for the National Standards of Bereavement Care following Pregnancy Loss and Perinatal Death [12].

### Learning opportunities

There were many presentations at the conference that were both thought provoking and informative, however, two pre-conference workshops stand out in particular. The first was the IMPROVE workshop (Improving Perinatal Mortality Review and Outcomes Via Education) [13]. The second a Pathology Workshop, devised by two perinatal pathologists (one from Ireland and one from Australia) specifically for volunteers from organisations who provide information and support to bereaved parents and that are primarily run by bereaved parents themselves.

As a model for learning, the IMPROVE workshop highlighted that for some senior doctors in training, this was the first formal input they received around perinatal mortality. While acknowledging that they had received most of their education and training from working on specialised pregnancy loss teams and thus felt well equipped to deal with stillbirths, it demonstrated how marginalised this area appears to be in medical training and education. In many ways, it is no surprise that research indicates that clinicians on the ground find pregnancy loss such a challenging area to work in [14]. How can a professional respond to someone in deep distress and provide best care if not adequately trained to do so? A recent systematic review and meta-analysis highlighted the need for evidence-based news delivery training in maternity services and effective frameworks that offer possibilities in this regard [15].

The perinatal pathology workshop also demonstrated real possibilities that exist for training and education, particularly by experienced practitioners on the ground. At this workshop two perinatal pathologists spoke openly and transparently about the nature of their work and the challenges inherent. Having the opportunity at ISA to learn from practice in different countries was really beneficial. It became very clear that we cannot expect best practice or quality service provision unless services are properly resourced and training and education are prioritised. The importance of voluntary organisations, upon which our public services depend to provide much needed support to bereaved parents, being fully informed and kept up to date with services and practices that parents may later have questions about, became very apparent. It was evident that this workshop had been very significant at the parents’ assembly when the need to lobby for fully resourced perinatal pathology services in Ireland was unanimously agreed.

The scientific programme of the conference was planned by the local organising committee (which included parent representatives) and was designed to ensure that the different streams would meet the needs of all delegates. The same conference abstract submission criteria applied for parents, clinicians and researchers and all conference presentation submissions were assessed using a blinded review process. Breakout sessions were specifically organised with parents in mind, ensuring a balance of presentations focusing on bereavement care and pregnancy after loss, with presentations around stillbirth investigations, audits and clinical practice. Much thought went into how to give voice to the experience of parents at the conference. With the encouragement of members of the PLRG, parents who expressed an interest were given support to submit an abstract to the conference. A number of these were accepted as poster presentations and aligned in focus with those submitted by researchers and clinicians. Support and advocacy organisations (primarily run by parents) from around the world were given space for their exhibition stands around the poster area, facilitating the exchange of ideas and networking possibilities for future collaborative work.

One bereaved parent gave an oral presentation and five parents presented a pre-recorded plenary documentary film, ‘When my baby died’, which was commissioned for the conference in collaboration with members of the PLRG. Recognising the need to represent the wide spectrum of people who are directly affected by stillbirth and pregnancy loss (e.g. mothers, fathers, siblings and extended family) and the differing circumstances under which stillbirth occurs, much consultation went into the making of this documentary, which was filmed in parents’ own homes prior to the conference. This group of parents were present in the audience as the film was screened. A hushed reverence descended across the auditorium as 400 people watched. One could not but be moved and awestruck at the courage and incredible resilience of these parents as their stories were shared.

This film affirmed the possibilities that exist when parents are supported in their commitment to research and the ensuing wisdom and insights that arise out of adversity. The importance of this being heard and valued as a starting point for research endeavours in the area of stillbirth, became abundantly clear. The appropriate timing to invite bereaved parents to be participants in research projects is something that is given much ethical consideration. While there were some parents who later said they found it emotionally challenging to attend some presentations and we came to realise the need to be conscious about this and provide reassurance for those who needed it, other parents who attended sessions in the more scientific streams, were clearly interested and engaged by what they had heard. Parents remarked that having the opportunity to hear about research and see the commitment of healthcare professionals to pursue best practice, gave them greater insight into the need for robust and scientific approaches to investigate the causes of stillbirth and an appreciation of the efforts being made to do this around the world. This reaffirmed the importance of parents having access to conferences and scientific research to witness this. Parents also recognised that it took courage as a practitioner to stand up in front of a group of parents who have experienced stillbirth and to be honest about the challenges of practice, the questions that remain unanswered and to invite commentary and questions. Practitioners too, had to be conscious of how they would present their research in a way that was accessible to an audience that included bereaved parents. However, it was clearly evident that it is only by doing exactly this, that mutual trust and respect can be built and a culture of support, collaboration and learning be sustained [5].

## Discussion

The collaboration and partnership between clinicians and bereaved parents to work together in the organising and running of an international scientific conference has provided much learning and insight for ongoing creative engagement to further the research and clinical care agenda in stillbirth prevention and care. The early involvement of parents in this process enabled an organic process to evolve and relationships of trust to form in what is a sensitive area of research and care. Many parents report that having the opportunity to share their bereavement experience for research purposes can be therapeutic in itself and say they want to be able to use their experience for the benefit of others and to contribute to developments in service provision. While ensuring that there is appropriate support for parents who participate is very important, our experience from this conference is that if carefully considered and meaningfully offered, participation by bereaved parents in scientific research has much to offer. Since the ISA conference, the Pregnancy Loss Research Group have expanded their research agenda to focus on and investigate areas of concern that were identified by parents. In addition, a newly established ‘Parents Forum’ is now being used in a consultative capacity as part of the implementation process for the new national maternity bereavement standards in Ireland. Cork University Maternity Hospital has also added a parent advocate to its bereavement committee as a result of the conference experience.

Four key messages arose from this endeavour that illustrate the richness of such collaboration, and support the growing expectation of meaningful patient / public involvement in major research projects.

### Key message 1 Partnership

Partnership was the hallmark of this conference. Drawing from an existing relationship and practice in the host Pregnancy Loss Research Group, this partnership between patient and healthcare professionals was an established one. This existing practice fostered a natural willingness to engage with parents as key partners and stakeholders in a scientific conference. The results of this partnership fostered a deeper awareness on the part of clinicians of the needs of parents and on the part of parents of the challenges and realities for clinicians working in this challenging area. Our hope as a committee is that the positive experience of partnership as experienced in this conference, will serve as an incentive for other scientific conferences to seek out the lived experience and views of patients as part of their research endeavour.

### Key message 2 The use of creativity as a unifying expression of grief and as a means to facilitate learning for parents and staff

The use of creativity in the making of mementos, provided a unifying and non-verbal method for both parents and staff, to symbolically represent and express the grief and loss associated with stillbirth. This tangible and creative expression facilitated the sharing of personal story and insight in a non-clinical and powerful way. Creativity also served as a valuable method to facilitate parents to process their learning from the conference. Having a supported platform to consider this and contribute their voice to the future research agenda around stillbirth prevention, was an important and empowering experience.

### Key message 3 The value of collaboration with global stakeholders in raising awareness about stillbirth

**Fig. 5:**
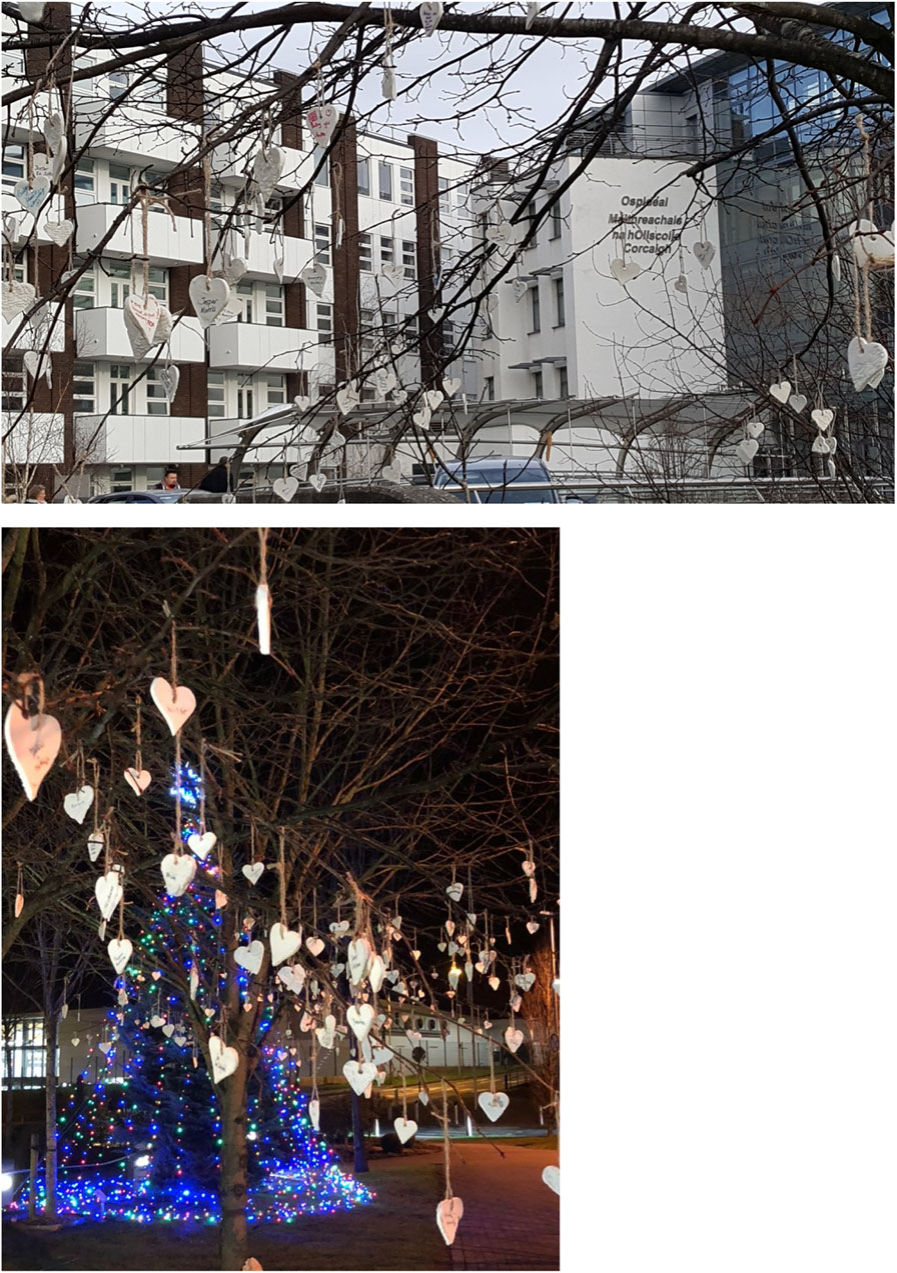
Hearts hanging outside CUMH – raising public awareness

This conference highlighted the value of collaboration with global stakeholders and the power of collective engagement about an important research area. In the renewed global call to redouble efforts to reduce preventable stillbirth, the increased global engagement with the topic of stillbirth through social media provided an important platform to raise public consciousness about stillbirth as a public health concern. In an on-going effort to raise awareness at a local level, after the conference was finished, the parent committee decided to hang the 600 inscribed hearts on tress outside Cork University Maternity Hospital, the largest maternity hospital in Ireland (Fig. 5). Once again, it was the commitment of parents, inspired by their learning from the conference that provided the impetus for this public awareness initiative. As members of the public passed, many stopped to look closer at the hearts and were visibly moved when they realised what they represented. Some even wondered if they were for sale! Our hope as a committee is that they might have started conversations so that stillbirth and pregnancy loss become part of the story of pregnancy and pregnancy related education and care.

### Key message 4 The importance of meaningful patient/public engagement in scientific research

The inclusion of meaningful patient/public engagement in clinical research can be challenging and as mentioned hitherto, has in some quarters, been labelled as ‘self-serving’. The development of genuine partnership and context as illustrated by this ISA conference demonstrates that this fosters a safe and trusting environment for the sharing of experience and insights in what is a deeply personal and sensitive area. This approach facilitated rich personal story and lived experience to be shared by clinicians and bereaved parents in a professional, mutually respectful and ultimately enriching way. This conference highlighted the inextricable connection between robust science and human experience and how they cannot be separated in the area of stillbirth. The impact of stillbirth as a personal and professional loss coupled with the challenge to identify and manage risk factors and causes of stillbirth, are equally worthy of scientific attention in the effort to reduce the individual toll and the incidence of preventable stillbirth worldwide.

## In conclusion – a lasting legacy

Undoubtedly, stillbirth is one of the most challenging areas of obstetric and midwifery practice and one of the most devastating experiences of bereavement for families. The creative and active collaboration between bereaved parents, clinicians, scientists and researchers in the planning and running of an international scientific stillbirth conference, highlighted the critical importance of partnership between those leading research and clinical care and bereaved parents. The experience of ISA 2017 suggests a number of key messages for meaningful patient public engagement with clinicians and researchers to improve bereavement care and to advance the global goal of reducing preventable stillbirths.

In a sensitive area of bereavement, the collaborative efforts of bereaved parents and healthcare professionals, highlighted the importance of addressing challenging areas of personal and professional loss together, and in so doing, forging critical and mutually accountable engagement to progress the research, clinical and bereavement care agendas, for the benefit of all.

## Abbreviations

CUMH: Cork University Maternity Hospital
IMPROVE: Improve Perinatal Mortality Review and Outcomes
INFANT: Irish Centre for Fetal and Neonatal Translational Research
ISA: International Stillbirth Alliance
PLRG: Pregnancy Loss Research Group
